# A Cardiac-Specific Robotized Cellular Assay Identified Families of Human Ligands as Inducers of PGC-1α Expression and Mitochondrial Biogenesis

**DOI:** 10.1371/journal.pone.0046753

**Published:** 2012-10-03

**Authors:** Matthieu Ruiz, Delphine Courilleau, Jean-Christophe Jullian, Dominique Fortin, Renée Ventura-Clapier, Jean-Paul Blondeau, Anne Garnier

**Affiliations:** 1 INSERM, U-769, Univ Paris-Sud, Châtenay-Malabry, France; 2 IFR141, CIBLOT platform, Univ Paris-Sud, Châtenay-Malabry, France; 3 BIOCIS, UMR 8076, Univ Paris-Sud, Châtenay-Malabry, France; Inserm, France

## Abstract

**Background:**

Mitochondrial function is dramatically altered in heart failure (HF). This is associated with a decrease in the expression of the transcriptional coactivator PGC-1α, which plays a key role in the coordination of energy metabolism. Identification of compounds able to activate PGC-1α transcription could be of future therapeutic significance.

**Methodology/Principal Findings:**

We thus developed a robotized cellular assay to screen molecules in order to identify new activators of PGC-1α in a cardiac-like cell line. This screening assay was based on both the assessment of activity and gene expression of a secreted luciferase under the control of the human PGC-1α promoter, stably expressed in H9c2 cells. We screened part of a library of human endogenous ligands and steroid hormones, B vitamins and fatty acids were identified as activators of PGC-1α expression. The most responsive compounds of these families were then tested for PGC-1α gene expression in adult rat cardiomyocytes. These data highly confirmed the primary screening, and the increase in PGC-1α mRNA correlated with an increase in several downstream markers of mitochondrial biogenesis. Moreover, respiration rates of H9c2 cells treated with these compounds were increased evidencing their effectiveness on mitochondrial biogenesis.

**Conclusions/Significance:**

Using our cellular reporter assay we could identify three original families, able to activate mitochondrial biogenesis both in cell line and adult cardiomyocytes. This first screening can be extended to chemical libraries in order to increase our knowledge on PGC-1α regulation in the heart and to identify potential therapeutic compounds able to improve mitochondrial function in HF.

## Introduction

In a highly oxidative tissue like the heart, mitochondria play an essential role to continuously adapt energy production to energy consumption for physiological functions. Muscle oxidative capacity and thus mitochondrial content largely depend on mitochondrial biogenesis and are linked to the activity of the transcriptional coactivator peroxisome proliferator-activated receptor γ coactivator 1α (PGC-1α) that is considered as the master regulator of energy metabolism [1]. In many tissues, an abnormal regulation of PGC-1α results in pathological consequences and we and others have shown that heart failure is tightly associated with a decrease in PGC-1α mRNA and protein expression in different rodent models [2], [3], [4] or in humans [5], [6] leading to energetic deficiency of the myocardium, while other studies pointed to a deficit of PGC-1α coactivating targets involved in mitochondrial biogenesis and mitochondrial DNA replication both in humans [7], [8] and animal models [8]. These alterations are accompanied by a deregulation of several mitochondrial pathways including, fatty acid utilization, mitochondrial biogenesis, and detoxification pathways. Accordingly, positive modulators of PGC-1α and its transcriptional cascade can be proposed as potential therapy [9], [10] to globally improve mitochondrial function and energy metabolism.

The mitochondrial genetic program requires activation of both the nuclear and mitochondrial genomes. Nuclear gene expression is under the control of transcription factors like the nuclear respiratory factors (NRFs), inducing the expression of the mitochondrial transcription factor A (Tfam). Tfam controls the replication and the transcription of the mitochondrial DNA. The coordination of both nuclear and mitochondrial genomes is orchestrated by PGC-1α which can stimulate the expression of nuclear-encoded transcription factors and co-activate them, thereby enhancing their transcriptional activity. PGC-1α expression and activity and mitochondrial biogenesis increase in different tissues in response to physiological stimuli that induce an increase in mitochondrial energy demand: endurance exercise training [11], [12], caloric restriction [13] or cold exposure [14]. These metabolic adaptations controlled by PGC-1α have been largely described in skeletal muscle, in brown fat and liver but with different profiles [15]. Exercise capacity correlates with PGC-1α expression in human skeletal muscle [16] but exercise training does not induce a relative increase in PGC-1α in the heart [17]. Cold exposure induces PGC-1α both in skeletal muscle and brown fat but not in brain and heart [14]. Resveratrol leads to an increase in PGC-1α activity in skeletal muscle and brown adipose tissue but again not in the heart or the liver [18]. Finally, calorie restriction increases PGC-1α expression in all studied tissues including the heart [19]. Collectively, this highlights a tissue and stimulus-specific regulation of PGC-1α that precludes extrapolating our knowledge of PGC-1α regulation from one tissue to the other.

In the heart, it is widely accepted that PGC-1α controls energy state and mitochondrial biogenesis, and the knowledge acquired with gain and loss of function experiments comfort this link. PGC-1α null mice exhibit a decrease in oxidative capacity accompanied by a switch in substrate utilization from fatty acid to glucose [20], [21]. This mitochondrial dysfunction resulting from a decrease in mitochondrial biogenesis was associated with the development of a cardiomyopathy and with an increase in heart failure markers and a reduction in fractional shortening [22]. Identification of activators of PGC-1α expression and mitochondrial biogenesis in adult cardiomyocyte is thus necessary to develop a new therapeutic strategy to counteract the metabolic failure of the failing heart.

Thus, to identify new inducers of PGC-1α expression in a cardiac background, we developed a “cardiac like” cell line gene reporter assay based on the expression of a secreted luciferase under the control of the human promoter of PGC-1α. We used this construction to screen a human ligand library with the following steps: a double primary screen based on luciferase activity and luciferase gene expression measurements, a dose-response validation, a toxicity test, a secondary validation in adult rat cardiomyocytes based on the expression of the endogenous PGC-1α and of its downstream targets, and finally a functional validation on mitochondrial respiration in treated H9c2 cells.

## Results

### Stable Cell Line Development and Characterization

Establishing a cell-based screening assay in adult rat cardiomyocytes is not straightforward. Adult cardiomyocytes are difficult to work with because they do not divide, they cannot be used for more than 48 hours in culture, the yield of cell dissociation is not sufficient to pretend to a robotized assay and finally because these cells are not transfectable and need adenoviral infection [23]. In addition, the use of primary cells would necessitate a large number of animals that raises an ethical issue. A screening assay based on endogenous PGC-1α mRNA expression was described for satellite skeletal muscle cells [24]. However such a strategy is not applicable due to the absence of satellite cells in cardiac muscle. We thus selected a myoblast cell line the H9c2 that is derived from embryonic BD1X rat heart tissue. This cell line was chosen because it is the only “ventricular-like” cell line that is suitable for a robotized assay. Moreover these cells are easily transfectable and allow the establishment of a stable cell line.

**Figure 1:**
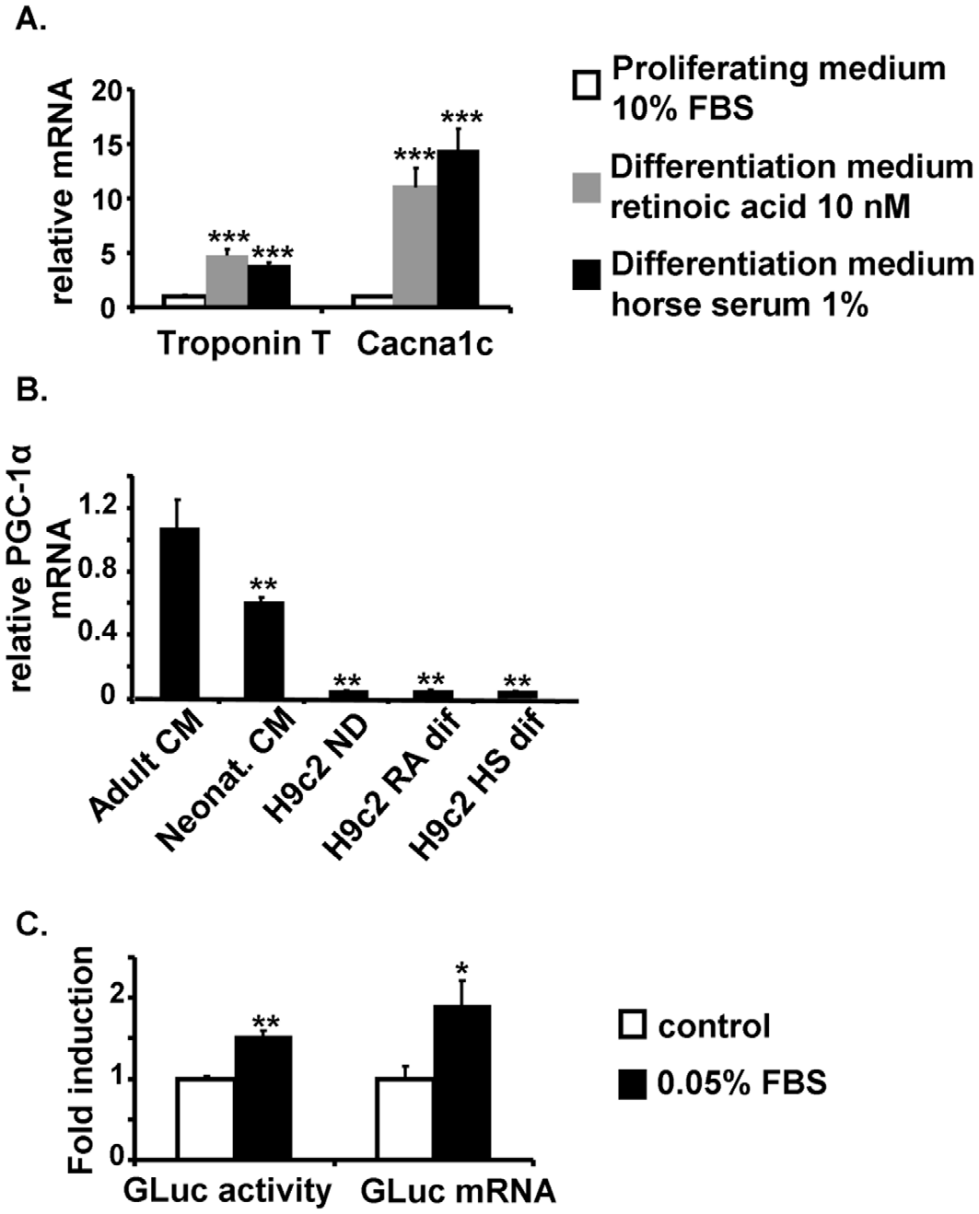
Characterization of H9c2 cell line. Differentiation of H9c2 cells with 1% horse serum (HS) for 6 days induced an increase in cardiac marker expression. (**A**) mRNA levels of Cacna1c and cardiac Troponin T normalized to geNorm after differentiation for 7 days with 10 nmol/L retinoic acid (RA) +1% Fetal Bovine Serum (FBS, grey) or by 1% HS (black) compared to control cells (white). (N = 6 independents culture). ***P<0.005 relative to proliferative medium condition (**B**) qPCR results expressed in ΔΔCT normalized to TBP as internal control to compare PGC-1α mRNA expression in different cell types: adult rat cardiomyocytes (CM), neonatal rat CM, H9c2 non differentiated cells (ND) or differentiated with RA or HS. (N = 5–7 independent cultures) (**C**). 0.05% FBS induced an increase in both Gaussia luciferase (GLuc) activity and GLuc mRNA expression in HS differentiated H9c2 cell line stably transfected with PGC-1α promoter/GLuc. (N = 6 independent cultures). *P<0.05, **P<0.01 relative to control.

H9c2 cells were maintained in the proliferative state in the presence of 10% FBS and proliferation was stopped by reducing serum concentration. Differentiation in a “cardiac like” phenotype was induced either in low FBS culture medium supplemented with retinoic acid (RA) [25] or with low horse serum culture medium [26]. After 6–7 days in the differentiation media, the gene expression of cardiac phenotype markers like cardiac troponin T (TnT) and cardiac (α_1C_) L-type Ca^2+^ channel (Cacna1c) was measured. A 5- and 15-fold increase in the expression of TnT and Cacna1c respectively was observed in the two differentiation media (Figure 1A). This cell line presented however a very low endogenous PGC-1α mRNA content compared to adult or even neonatal cardiomyocytes (Figure 1B). We thus developed a gene reporter cell line for PGC-1α expression to get a more robust and specific assay.

For this purpose, the proximal 2.7 kb human promoter of PGC-1α was amplified and cloned in a GLuc basic vector. This length of promoter was chosen according to the literature because it integrates all the responsive elements actually known to be involved in PGC-1α regulation in skeletal muscle or adipocytes [27], [28]. Studies with the gene reporter technology classically use a vector coding for Renilla or Firefly Luciferase that necessitates a step of cell lysis before the measurement of the luminescence production. The originality of GLuc is based on the presence of a secretion signal sequence in the cDNA coding for GLuc [29]. This secretion allows to measure luciferase activity in the culture medium thus allowing cells to be kept alive for GLuc mRNA measurement or viability assay in the same well.

The stable cell line was established using Fugene HD technology and geneticin to achieve a selective pressure. After 15 days of selection, basal GLuc activity was maintained for several passages. To take into account chromatin modifications and to promote multiple sites of integration in H9c2 genome, we chose to work with a pool of selected cells instead of a stable clone.

In order to select a positive control, different known inducers of PGC-1α expression in skeletal muscle cells or adipocytes as for example forskolin, were tested but failed to induce GLuc expression in our cardiac model. The functionality of our construction PGC-1α/GLuc was thus validated with FBS which has emerged as a positive control because it contains a mixture of growth factors and hormones. A 0.05% FBS was chosen as it gave the maximal induction of G-Luc activity with the minimal FBS concentration. After differentiation, 0.05% FBS was able to induce an 80% increase in GLuc activity under the control of the PGC-1α promoter and a two fold induction of GLuc mRNA expression (Figure 1C). Taken together, these results show that we have established a functional stable “cardiac-like” cell line expressing a reporter gene under the control of the human PGC-1α promoter.

### General Results of the Primary Screening and Identification of Positive Hits

**Figure 2:**
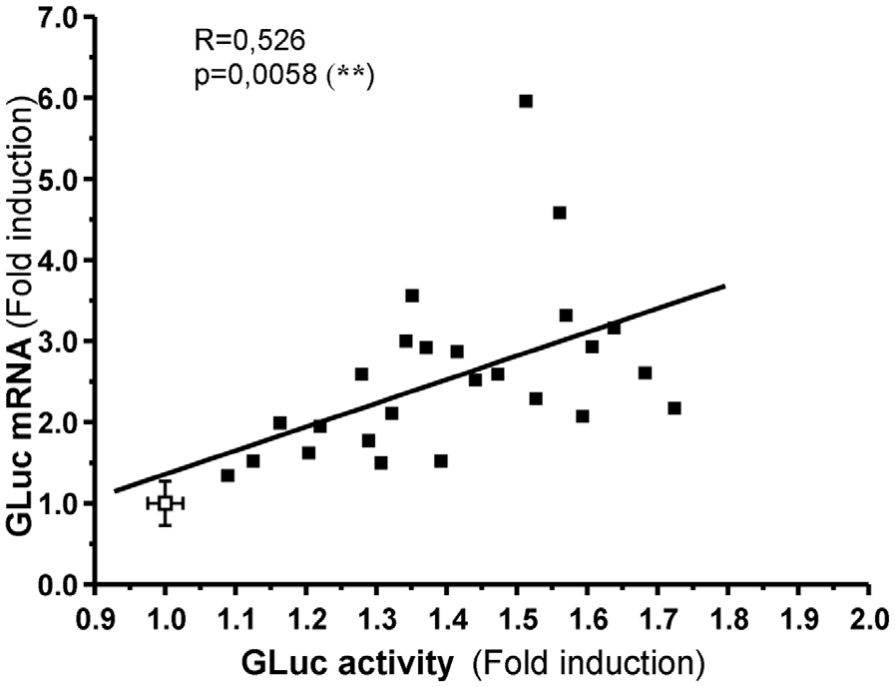
Correlation between Gaussia luciferase (GLuc) activity and gene expression for positive hits resulting from the primary screen. Twenty five compounds were identified as positive hits (black squares). Control values (n = 6) were expressed as mean ±SEM (white square).

**Figure 3:**
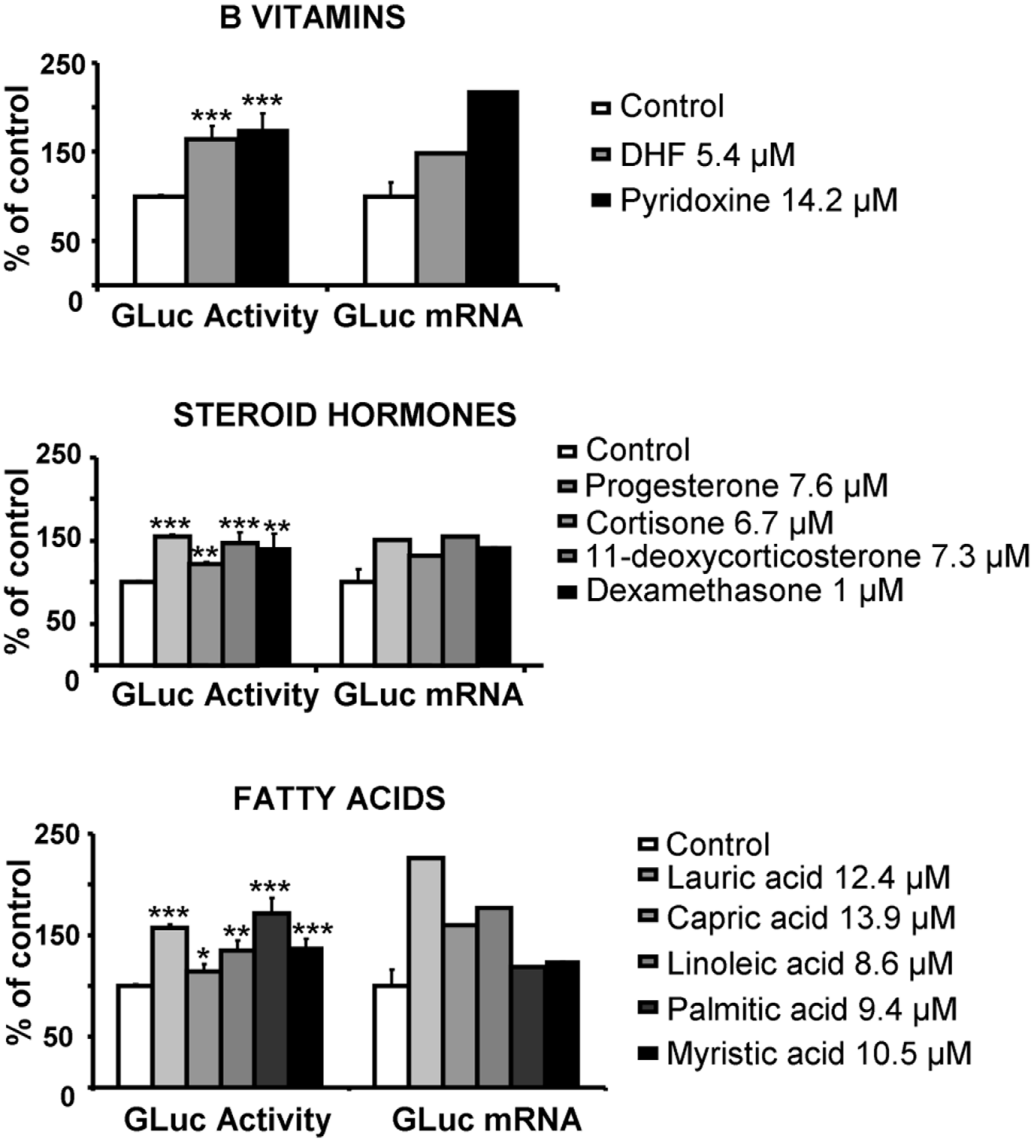
Principals selected hits families based on Gaussia luciferase (GLuc) activity and gene expression. This screen identified three main families inducing PGC-1α promoter activation: B vitamins, steroid hormones and fatty acids. (N = 4 for GLuc activity, 1 for mRNA expression). *P<0.05, **P<0.01, *** p<0.005 relative to control.

For the first screening assay, we used a robotized cellular assay (RCA) method to screen a selection of compounds from the LIGENDO library and of potential interest. This library is composed of large molecular diversity of metabolite-like compounds. Eighty compounds were thus tested in the first-round screen for GLuc activity (global results are presented in Table S1). Then the second-round screen was performed to measure GLuc mRNA expression. Molecules were defined as positive hits for PGC-1α activation when GLuc activity was statistically different from that of controls and was associated with an increase in GLuc mRNA expression above a threshold of 1.3. The correlation between GLuc activity and mRNA expression (Figure 2) showed that among the 80 selected compounds, 25 could be defined as activator hits (p<0.01) (Table S3). Eleven molecules belonged to three major families of compounds: B vitamins, steroid hormones and fatty acids, and presented a range of dynamic response from 1.13 to 1.72 for GLuc activity and from 1.52 to 4.58 for GLuc mRNA (Table 1). None of these molecules induced cellular toxicity. Figure 3 illustrates the response of representative human ligands of each family with a positive effect on GLuc activity and GLuc mRNA expression. On the figure are indicated the concentrations calculated from the amount and molecular weight of the compounds used in the first screen.

**Table 1:** General results of the primary screening and validation.

Family	N	GLuc activity	P value	GLuc mRNA	Toxicity	Confirmed positives
**Vitamins**	2	1.60–1.72	<0.05	2.17–2.93	0 of 2	1 of 2
**Steroid hormones**	4	1.2–1.5	<0.05	1.52–2.52	0 of 4	1 of 4
**Fatty acids**	5	1.13–1.68	<0.05	1.52–4.58	0 of 5	2 of 3
**Others**	14	1.22–1.63	<0.05	1.34–5.96	0 of 16	
**Total**	**25**	**1.13–1.72**	**<0.05**	**1.34–5.96**	**0 of 27**	**4 of 9**

N indicates the number of positive hits in respective families. Gaussia Luciferase (GLuc) activity and gene expression were expressed as fold induction compared to the average signal of control wells. P value was based on luminescence assay for GLuc activity (n = 4). Toxicity was evaluated by Alamar Blue assay and DNA content. The hit selection resulting from the primary screen was validated in a repeat of the screen including a dose-response (confirmed positives).

**Figure 4:**
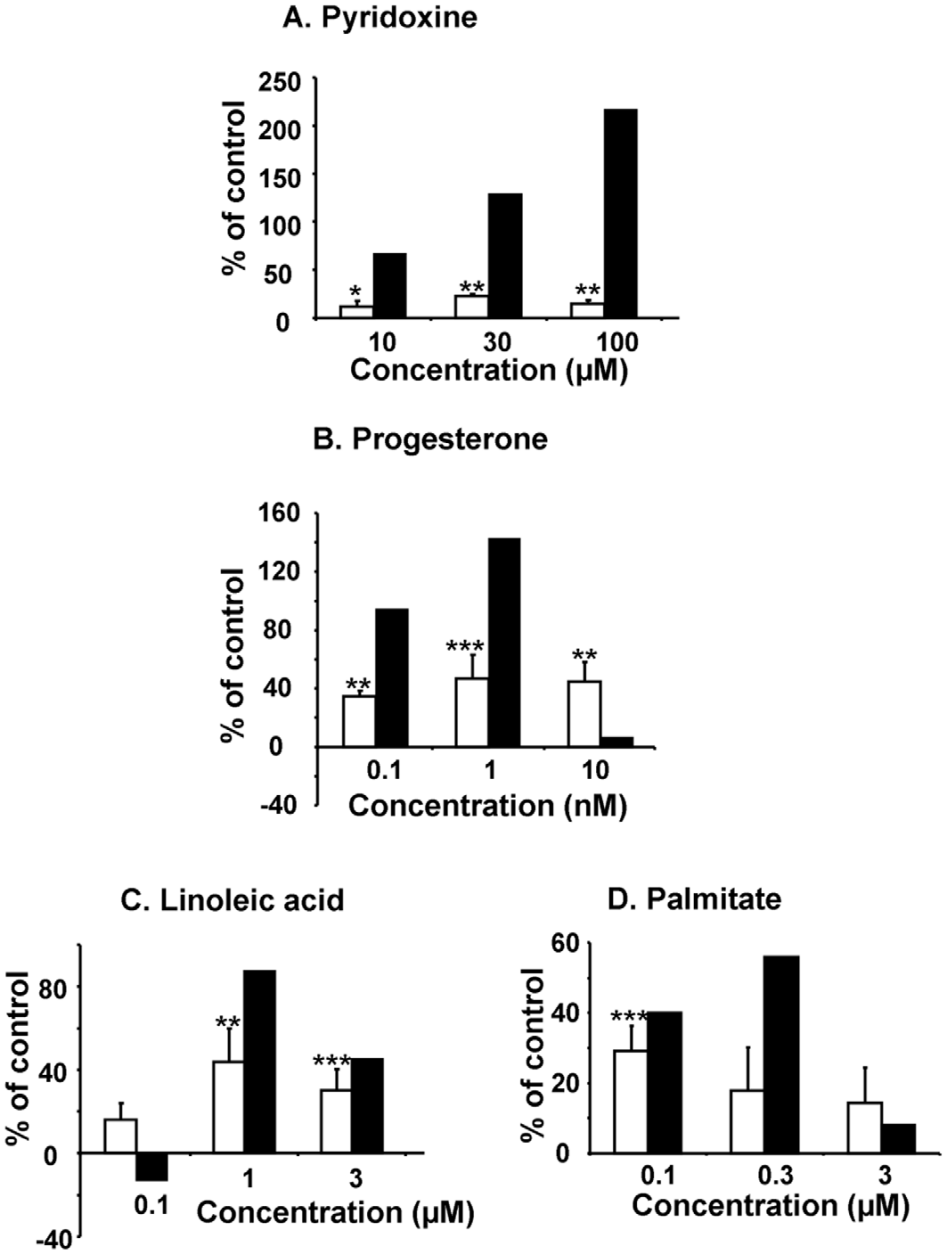
Validation of the positive hits selected from the first screen. GLuc activity (open bars, N = 4) and mRNA expression (black bars, N = 1) were measured following 9 hours of treatment with pyridoxine (Figure 5A), progesterone (Figure 5B), linoleic acid (Figure 5C) and palmitate (Figure 5D) at different concentrations. **P<0.01, ***P<0.005 relative to control.

To validate these findings, a new plate containing some of these positive compounds from commercial provenance was generated and different doses was tested (Figure 4). For each compound and each dose, and based on either GLuc activity or gene expression, we confirmed 1 of 2 vitamins: pyridoxine at the dose of 100 µM (Figure 4A), 1 of 4 steroid hormones: progesterone at 1 nM (Figure 4B) and 2 of 3 fatty acids: linoleic acid and palmitate at 1 µM (Figure 4C) and 100 nM (Figure 4D) respectively. These four compounds were then chosen to perform experiments in adult rat cardiomyocytes at the indicated concentrations.

**Figure 5:**
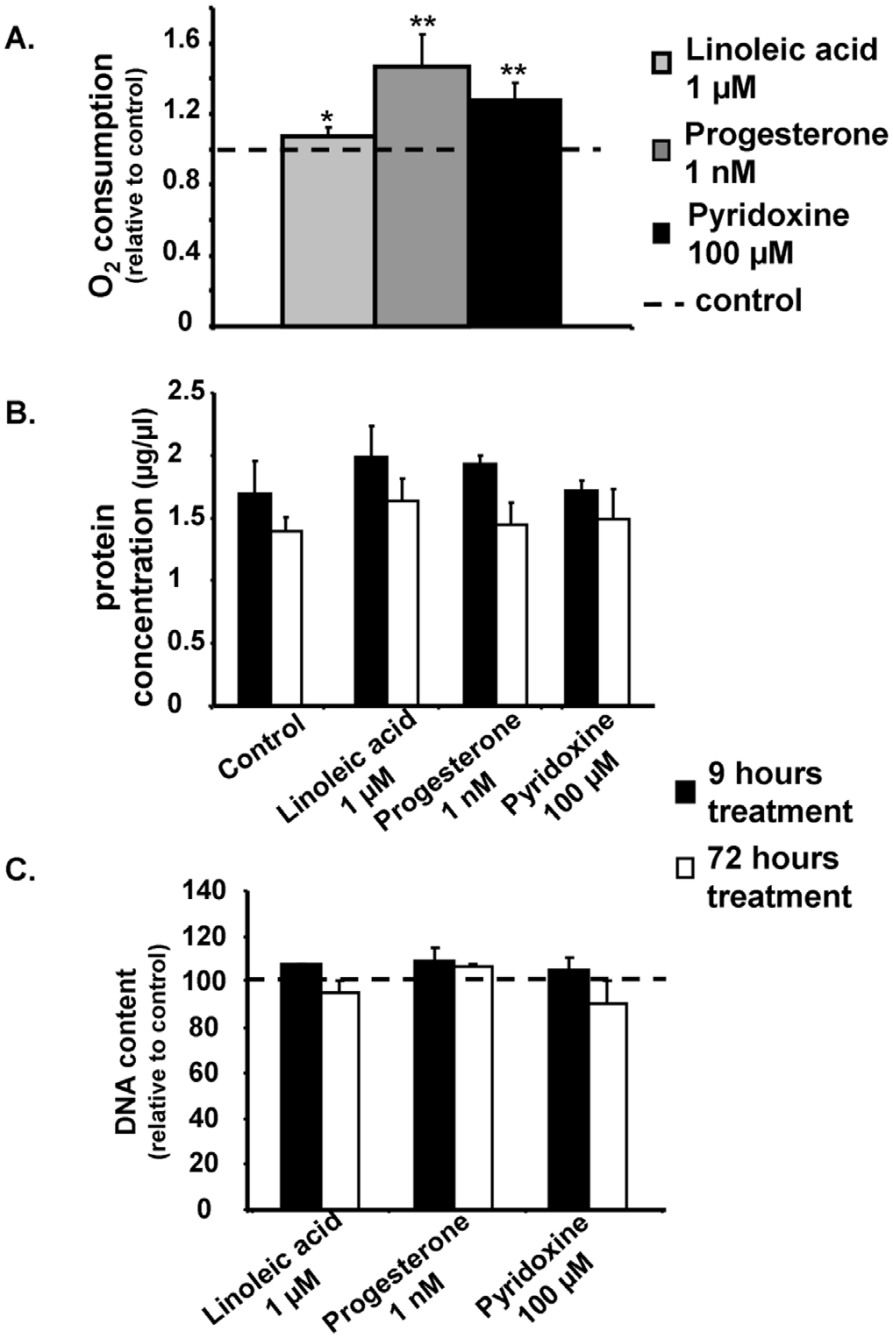
Functional validation of mitochondrial biogenesis by the measure of cellular respiration in treated-H9c2 cells. Following 72 hours stimulation with linoleic acid (1 µM, white), progesterone (1 nM, grey) and pyridoxine (100 µM, black) respiration rate was significantly increased compared to non stimulated cells (Figure 6A). Results are normalized to total protein content and expressed in % of control (N = 4 independent cultures). Figure 6B and 6C illustrated total protein and DNA contents respectively in H9c2 cells treated for 9 hours or 72 hours with the three compounds. *P<0.05, **P<0.01 between treatment group and control (dotted line).

**Figure 6:**
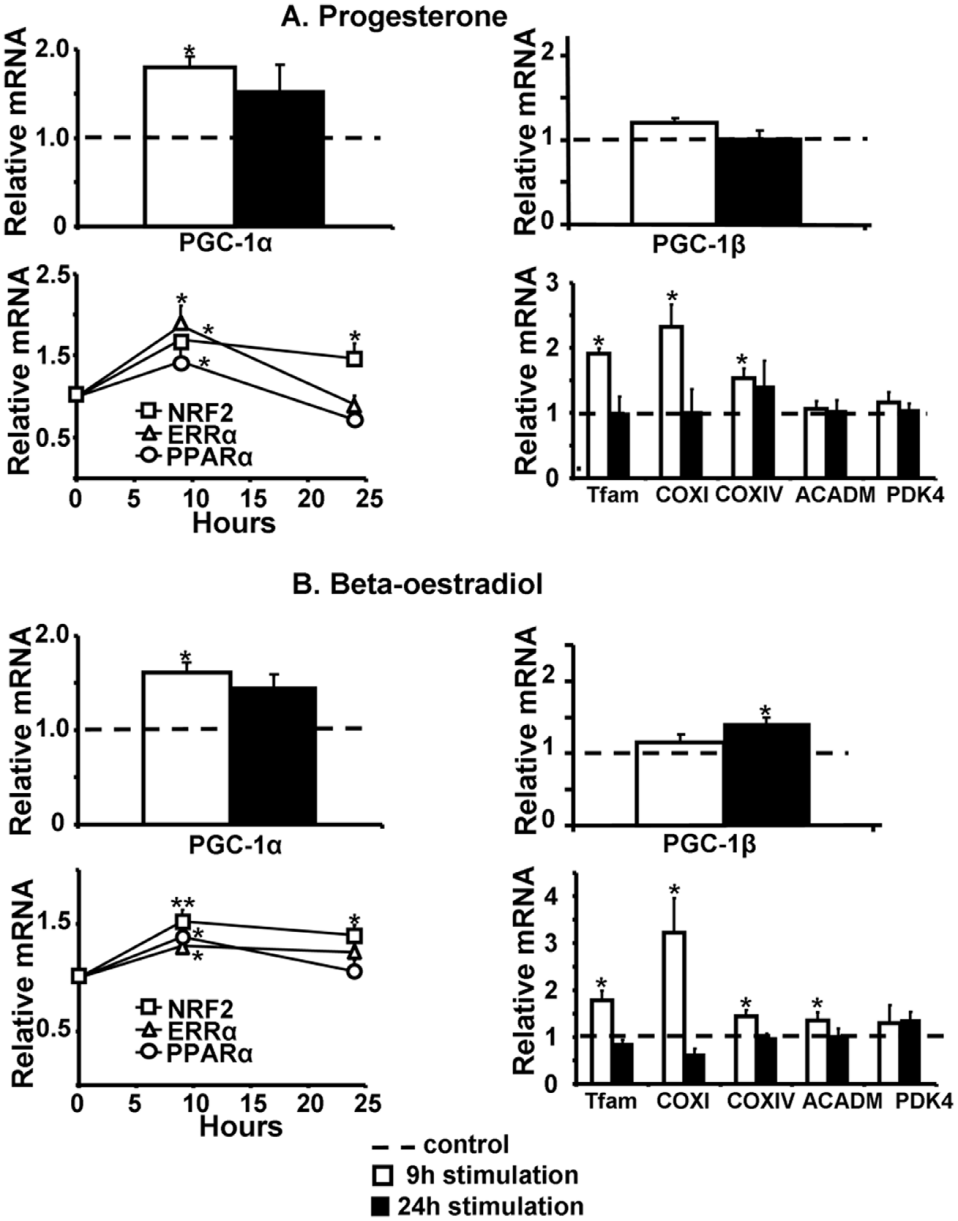
Validation of steroid hormones in adult rat cardiomyocytes. Nine (white columns) and 24 (black columns) hours stimulation with 1 nM progesterone (A) or 0.1 nM beta-estradiol (B) led to increased mRNA expression. RT-qPCR analysis of genes involved in mitochondrial biogenesis, normalized to TBP as internal control: PGC-1α (top left), PGC-1β (top right), transcription factors (bottom left): NRF2 (square), ERRα (triangle) and PPARα (circle) and downstream targets like Tfam, COXI and COXIV (bottom right). (N = 4–5 independent cultures). *P<0.05, **P<0.01 between treatment and control (dotted line) groups.

### Functional Confirmation in H9c2 Cells on Cellular Respiration

To examine whether the activation of mitochondrial biogenesis genetic program by the selected compounds may lead to a physiological increase in oxidative phosphorylation, total respiration was measured (Figure 5A) in permeabilized cells. After a 3-day treatment with linoleic acid, pyridoxine or progesterone, cellular O_2_ consumption was measured. Linoleic acid led to a significant 10% increase in total respiration, while progesterone and pyridoxine induced a 40% and 30% increase respectively.

To ensure that our positive hits were not able to induce growth or cell proliferation, total protein (Figure 5B) and DNA contents (Figure 5C) were measured after 9 or 72 hours treatment with linoleic acid, pyridoxine or progesterone. No significant difference was observed compared to non treated cells for total protein content as well as for DNA content. Thus, these results suggested that the effects of these positive hits on GLuc activity and mRNA expression in the first screen (9 hours treatment) or on cellular respiration (72 hours treatment) were not related to increase in cellular activity.

### Secondary Validation in Adult Rat Cardiomyocytes: mRNA Expression of Endogenous PGC-1α and its Downstream Targets

**Figure 7:**
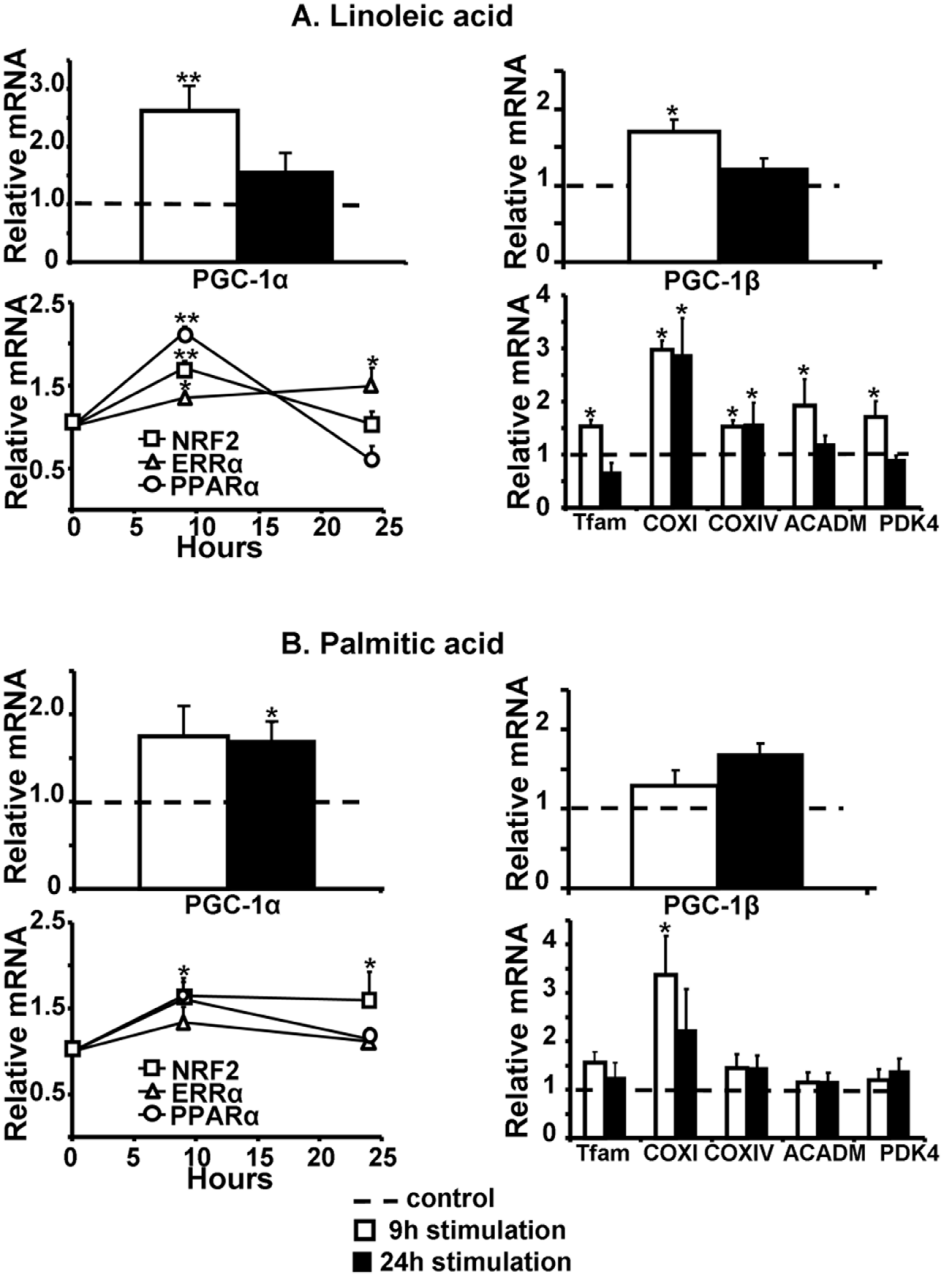
Validation of fatty acids in adult rat cardiomyocytes. Nine (white columns) and 24 (black columns) hours stimulation with 1 µM linoleic acid (A) or 100 nM palmitic acid (B). (N = 4–5 independent cultures). Legend is as in Figure 7.

**Figure 8:**
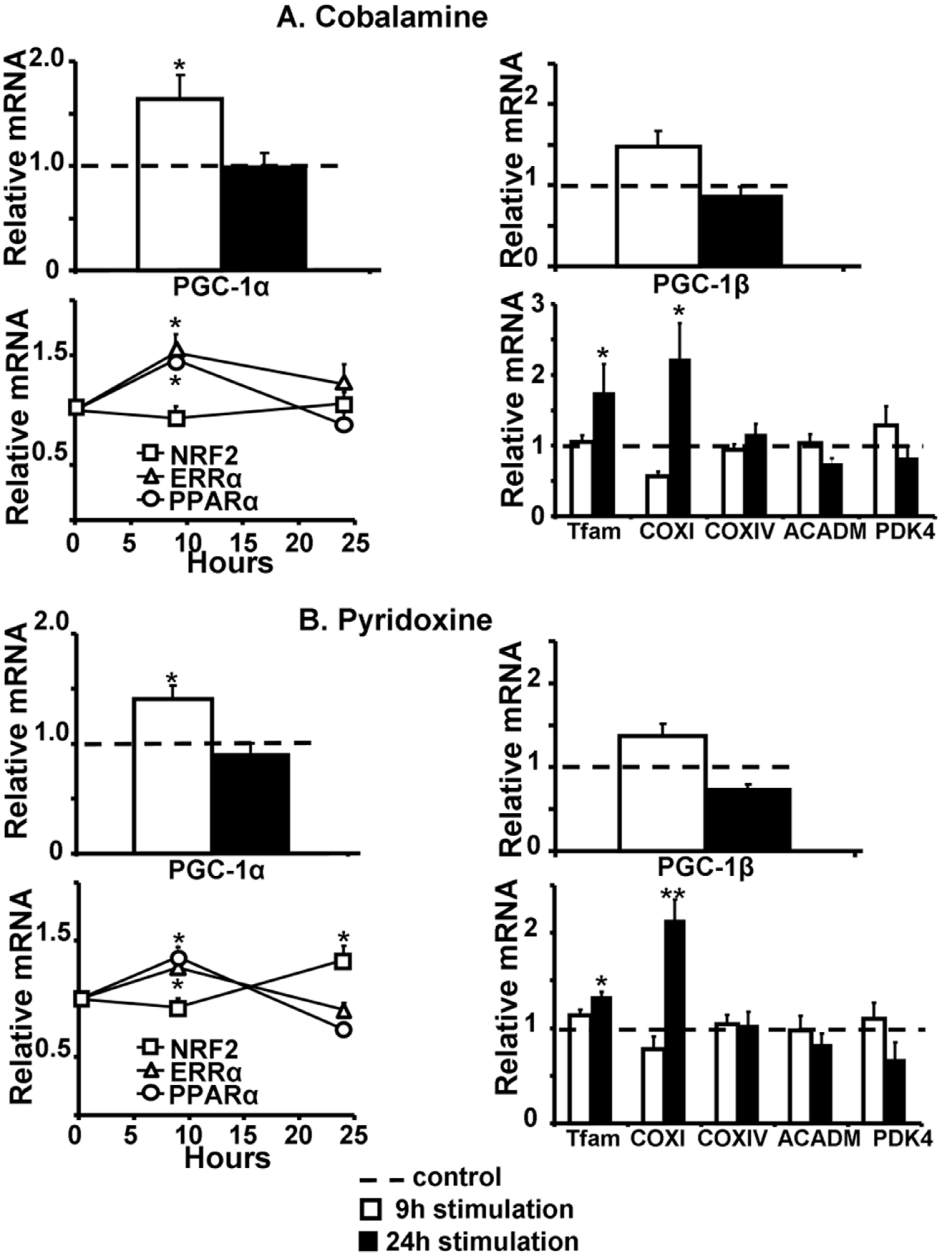
Validation of B vitamins in adult rat cardiomyocytes. Nine (white columns) and 24 (black columns) hours stimulation with 100 µM pyridoxine (A) or 30 µM cobalamine (B). (N = 4–5 independent cultures). Legend is as in Figure 6.

To ascertain the relevance in adult cardiomyocytes of the selected hits, obtained using the reporter gene for PGC-1α expression in the “cardiac-like” cell line, their effectiveness was assessed on the endogenous PGC-1α mRNA expression of adult rat cardiomyocytes treated for 9 or 24 hours. As β-estradiol is known to be an activator of PGC-1α [30] and deficiencies in cobalamine (B-vitamin) are associated with a decrease in mitochondrial mRNA content suggesting a link between cobalamine and mitochondrial biogenesis [31], we included beta-estradiol and cobalamine in this validation test. Endogenous PGC-1α transcription was increased with all selected molecules (Figures 6, 7 and 8). To check whether this transcriptional effect led to an increase in PGC-1α activity, mRNA expression of its downstream targets was measured. ERRα and NRF2, two factors activated and co-activated by PGC-1α and classically used as markers of PGC-1α activation were increased. This activation induced the downward expression of Tfam and of COXI and COXIV, two subunits of the complex IV of the mitochondrial respiratory chain encoded respectively by the mitochondrial and the nuclear genomes, evidencing a global increase in mitochondrial biogenesis.

Steroid hormones led to a rapid but transient activation of PGC-1α and its downstream targets (Figures 6A and 6B respectively for progesterone and beta-estradiol). Fatty acids, and notably linoleic acid, exhibited the strongest effect that was maintained at 24 hours (Figures 7A and 7B). B vitamins effect appeared delayed as PGC-1α was induced after 9 hours stimulation but totally returned to control at 24 hours stimulation, whereas mitochondrial biogenesis markers were activated between 9 and 24 hours (Figures 8A and 8B). Because PPARα is known to be activated by PGC-1α and fatty acids, its expression was also measured. Linoleic acid induced a huge increase in PPARα and its known targets like ACADM and PDK4 (Figure 7A). In all conditions, LDH activity measured in the supernatant confirmed the non-toxicity in cardiomyocytes (data not shown).

**Figure 9:**
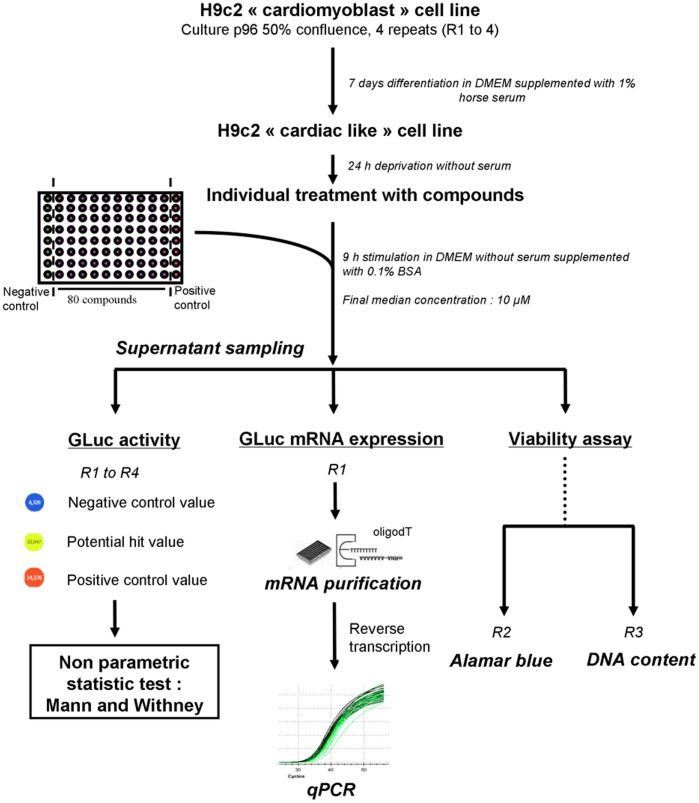
Flow chart describing the Robotized Cellular Assay (RCA).

 Taken together, these data indicate that treating cells with these ligands induces a gene program involved in mitochondrial biogenesis and that the results obtained with our reporter assay in H9c2 cell line could be extrapolated to adult cardiomyocytes. Moreover the 2.7 kb promoter fragment appears to have a sufficient length to recapitulate endogenous gene activation.

### Effect on PGC-1β Transcription

Because PGC-1β, the second member of the PGC-1 family, appears to have a large overlapping role with PGC-1α expression and because its expression is also decreased in heart failure [10], [32], we tested whether compounds able to activate the α isoform will be also effective on the β isoform. Among steroid hormones, only β-estradiol presented a delayed positive effect on PGC-1β expression at 24 hours while PGC-1α expression was already increased at 9 hours and maintained at 24 hours (Figure 6B). On the contrary, progesterone did not induce PGC-1β expression and thus appeared to be PGC-1α specific (Figure 6A). Only linoleic acid activated both PGC-1α and PGC-1β expression (Figures 7A).

## Discussion

In this study we developed a cellular reporter gene assay to find specific activators of PGC-1α in a cardiac background. This screening assay allowed preselecting a set of hits able to induce GLuc activity and mRNA expression in response to activation of the human PGC-1α promoter. With the ability of GLuc to be secreted, in the same test we could perform viability assay to select compounds with low toxicity. This strategy was largely validated by its effective transposition in adult rat cardiomyocytes. Being able to select potential cardiac activators of PGC-1α offers great possibilities, in particular to get insights into the physiological modulation of PGC-1α in adult heart and in a long term strategy to be able to select drugs with potential pharmaceutical action in a clinical perspective.

The H9c2 cell line, by its ability to acquire a “cardiac like” phenotype allows assessing the specificity of PGC-1α regulation in the heart. However this robotized cellular assay is not really suitable for high throughput screening due to the need of quadruplicate of GLuc activity measurements for statistical robustness. Indeed, due to the weak dynamic range of induction, it was not possible to establish an acceptable Z’ factor, we thus adapted the test with a post-hoc statistical analysis on four repeated measurements. Coupling GLuc mRNA expression to its activity allowed strengthening the reliability of the test. The strategy was finally validated in adult rat cardiomyocytes where all selected compounds led to PGC-1α activation, the induction of its downstream targets, and upregulation of mitochondrial biogenesis. For example, the increased expression of a nuclear encoded subunit of complex IV of the respiratory chain, COXIV and of the mitochondrial transcription factor Tfam, was linked to NRF2 and ERRα induction, while the mitochondria-encoded COXI subunit was linked to Tfam induction [33].

We chose at first to screen a selection of compounds from a human ligand library to validate our strategy and to identify physiologically relevant activators of PGC-1α expression in a cardiac background. From this first screening, three families could be identified.

Among fatty acids, linoleic acid appeared as an effective inducer of all mitochondrial biogenesis markers by activating both ERRα and NRF2 expression. In addition a strong activation of PPARα expression leading to the induction of its target genes like ACADM and PDK4 was observed. Palmitate was less efficient than linoleic acid in activating mitochondrial biogenesis. This family led to the rapid induction of mitochondrial biogenesis after 9 hours of stimulation. Interestingly, the expression of PGC-1β was also notably increased by linoleic acid. Long chain fatty acids are the preferential agonists of the nuclear receptor PPARα [34]. They increase PPARα expression, and that of its target genes involved in the binding, transport and oxidation of fatty acid as well as mitochondrial biogenesis [35], [36]. In addition, a recent study described a link between fatty acid content, PPARα activation and up-regulation of PGC-1α and β, leading to mitochondrial biogenesis in the heart [37] suggesting that PPARα activation could be responsible for PGC-1α and β induction. Interestingly, a cardioprotective effect of fatty acids is increasingly reported both in experimental models [38], [39] of heart failure and human studies [40], [41]. Moreover, we recently showed that the cardioprotective effect of resveratrol was associated with increased expression of the PPARα axis, increased fatty acid oxidation and mitochondrial respiration [42]. It can thus be proposed that the beneficial effects of fatty acids on mitochondrial biogenesis and function may take part in these cardioprotective effects.

Different steroid hormones were identified as potential activators of PGC-1α expression and mitochondrial biogenesis in the heart. Interestingly, the expression of PGC-1β was also increased by β-estradiol but later than PGC-1α. Progesterone failed to induce PGC-1β expression, proposing this molecule as the only compound specific for PGC-1α in this study. These results suggest a differential regulation of the two co-activators depending on the stimulus. These last years, a concept of sex and gender difference in the outcome of cardiovascular diseases highlighted the protective effect of female hormone impregnation. Indeed, women are better protected against the development of myocardial failure than men and notably under pressure overload [43], [44]. Interestingly, in a model of heart failure in mice, a higher expression of a panel of genes controlling mitochondrial function including PGC-1α was observed in females than males and a binding site for ERα was identified in the PGC-1α promoter [30]. Administration of 17β-oestradiol after trauma-hemorrhage restores depressed cardiac function by an ER-mediated up-regulation of PGC-1α [45], suggesting that ERβ-dependent PGC-1α-activation is involved in cardioprotection [46]. The possible involvement of progesterone in mitochondrial biogenesis is poorly documented. Progesterone was shown to regulate some genes of oxidative metabolism in brain and brown adipose tissues [47].

The third identified family was B vitamins, for which we observed a global increase in mitochondrial biogenesis markers, primarily linked to PGC-1α stimulation. Moreover, activation of Tfam and COXI seemed to be linked to ERRα expression. The present result showing a positive effect of B vitamins on mitochondrial biogenesis appears to be an original finding as nothing is known in the literature. However, it can be speculated that B vitamins like cobalamine or pyridoxine being methyl donors they are needed for the remethylation of homocysteine into methionine. A deficiency in B vitamins has been associated with a decrease in methionine conversion in favor of homocysteine accumulation called hyperhomocysteinemia (HHCY) [48], [49]. In cardiovascular diseases, HHCY represents a new recognized risk marker because its increase is associated with heart failure severity [50], [51]. B vitamin deficiency can induce mitochondrial disorders [31]. Hepatic cobalamine deficiency leads to accumulation of polycistronic mitochondrial RNAs and decreased mitochondrial mRNA content [52]. In a cellular model of Parkinson, treatment with high doses of B vitamins inhibits the toxicity induced by rotenone, an inhibitor of complex I of the respiratory chain. The amelioration of mitochondrial dysfunction is associated with an increase in PGC-1α expression [53]. Finally, in a model of cardiomyopathy induced by methyl donor deficiency, an imbalance in methylation/acetylation of PGC-1α leads to a decrease in its activity [54]. Therefore, B vitamins supplementation would improve cardiac function through mitochondrial function depending on PGC-1α activation.

We have thus identified three families of compounds able to activate PGC-1α expression in a cardiac-like cell line that were validated in adult cardiomyocytes. Interestingly these activators were able to activate the transcriptional cascade of mitochondrial biogenesis among which ERRα that was also identified to play a pivotal role in the pathophysiology of heart failure [7], [8]. Finally, because H9c2 cells or adult cardiac myocytes produce/consume far less high energy as *in vivo*, and because of the interference with other cell types and organs, these targets should be further validated *in vivo* under physiological condition and heart failure.

We describe here the effectiveness of a cardiac background robotized cellular assay for PGC-1α expression. By screening a human ligand library this assay allowed to identify new families of human ligands able to induce PGC-1α gene expression and mitochondrial biogenesis. This assay can be used to screen chemical libraries to find compounds able to improve mitochondrial function in heart failure. This offers new perspectives to identify new pathways involved in mitochondrial biogenesis regulation in the heart and in the future to identify a potential metabolic therapy of heart failure.

## Methods

### Ethics Statement

All experiments were performed in conformity with the European Community guiding principles in the care and use of animals (Directive 2010/63/EU of the European Parliament). Authorizations to conduct animal experiments were obtained from the French “Ministère de l’Agriculture, de la Pêche et de l’Alimentation” (no. 92–284, June 27, 2007).

### PGC-1α Promoter Cloning and Plasmid Construction

A 2.7 kb fragment of the human PGC-1α promoter was amplified by PCR using human genomic DNA (Clontech). PCR primers were designed from the PGC-1α promoter sequence (Gen Bank Accession number BD 103728; sense: 5′- GAG TTG ACG AAG GGG TGA AA - 3′, antisense: 5′ - CAA CCA GCC CCT TAC TGA GA - 3′). This PCR fragment was first ligated into the pCR-XL-TOPO vector and then subcloned into the *EcoRV-* and *BHI-* digested sites of the pGLuc-basic vector containing the Gaussia Luciferase (GLuc) reporter gene (New England Biolabs). This fragment (p2665) contains 2649 base pairs 5′- and 16 base pairs 3′- relative to the transcription start site and was verified by sequencing.

### Cell Culture and Treatment

The rat cardiomyocyte derived H9c2 cell line was obtained from ATCC (CRL-1446; passage 13) and grown in complete medium: Dulbecco's modified Eagle’s medium (DMEM) with high glucose supplemented with 10% fetal bovine serum (FBS) and antibiotics (100 units/mL penicillin and 100 µg/mL streptomycin (PS)) at 37°C with 5% CO_2_. When cells reached 50% confluence, they were switched to the differentiation medium: DMEM containing 1% horse serum (HS) and antibiotics for 7 days. The medium was changed every 2 days to obtain a differentiated “cardiac like” cell line. The cell line was used between passage 20 and 30.

### Transfection and Selection of Stably Transfected Cells

Transfection of H9c2 cardiomyoblasts with the PGC-1α promoter/GLuc DNA (PGC-1α/Gluc) was performed using Fugene HD according to the manufacturer’s instructions (Roche Diagnostics). Cells were cultivated in DMEM supplemented with 10% FBS and geniticin at 500 µg/ml. Geneticin-resistant colonies were selected 10–12 days after transfection and propagated. The obtained clones were grown in the same medium supplemented with geniticin at 200 µg/ml to maintain sufficient selective pressure.

### General Procedure of the Robotized Cellular Assay (RCA)

Before screening, cells were plated into 96-well plates at 2000 cells/well in quadruplicate with a microfill distributor (BioTek instrument) and cultivated in complete medium to obtain 50% confluence. After 7 days in differentiation medium, cells were deprived of serum for 24 hours. Stimulation was performed in DMEM without serum but supplemented with 0.1% BSA.

Six µl of each compound, were pin-transferred into each well to obtain a final median concentration of 10 µmol/L using a Biomek FX (Beckman Coulter) equipped with a 96-pin array. Each well was treated individually with one molecule. The left column was filled with DMSO as negative control. Nine hours later, the culture media from the four plates were transferred into a 384-well plate to measure GLuc activity for the first round screen. These 4 repeats of the experiments allowed to statistically validating our results with the non parametric Mann and Withney test. One plate was used for the secondary round screen consisting of mRNA extraction and qPCR measurement of GLuc expression. This double screening assay based on GLuc activity coupled to GLuc mRNA measurement allowed to define potential hits *i.e.*, PGC-1α activators. Two other plates were used to evaluate the toxicity of the molecules using the Alamar blue assay normalized to the DNA content.

A flow chart of our strategy is presented in Figure 9.

### Gaussia Luciferase Reporter Assay

Under the activation of the PGC-1α promoter upstream of the GLuc gene, this luciferase was produced and secreted and GLuc activity was directly measured in the cell culture medium using the Biolux GLuc Flex Assay kit (New England Biolabs) according to the manufacturer procedure in a plate reader (Envision Xcite, Perkin Elmer). In the absence of bovine serum albumin (BSA) supplementation, a fast degradation of the GLuc was observed. Thus, all stimulations were performed in the culture medium without FBS and supplemented with 0.1% BSA.

### RT qPCR Measurement

The H9c2 cells were washed twice with PBS and then lysed in 50 µl lysis buffer (QIAGEN). After 5 minutes incubation at room temperature, 40 µl cell lysate were transferred into a 96-well oligo(dT)-coated plate (QIAGEN) and incubated for 60 minutes at room temperature with constant stirring (100 rpm). The plate was then washed three times with the washing buffer and reverse transcription was performed in the same wells according to manufacturer’s instructions (BioRad) in 25 µl total volume. Five µl of this solution was transferred to a 96-well qPCR plate (BioRad) and 10 µl of buffer mix containing SYBR Green (BioRad) and PCR primers were added. For adult cardiomyocytes, standard procedures were used for total RNA extraction (Trizol reagent) and reverse transcription (iScript). Quantitative real-time PCR was performed by using a CFX96 PCR detection system (BioRad). The qPCR data were analyzed using the standard curve method for adult cardiomyocytes and the ΔΔCT method for GLuc mRNA expression and all other measurements. Data were normalized using geNorm for cell line comparison and to TBP for other measurements. The different primers used are listed in Table S2.

### DNA Content Measurement

The DNA content was determined as an index of the cell number in each well. Cells were lysed in 50 µl 0.1 N NaOH, 0.1% triton buffer for 45 minutes at 4°C. Twenty µl of each lysate was transferred to a black 96-plate in duplicate. To quantify double-strand DNA (Invitrogen), the DNA staining PicoGreen was diluted with Tris-EDTA (10/1 mM) and 100 µl of this solution were added in each well. Fluorescence of PicoGreen was detected using a Fluorometer (Envision Xcite, Perkin Elmer), with excitation at 485 nm and emission at 520 nm. DNA quantification was performed using a standard curve of salmon sperm DNA at known concentrations.

### Alamar Blue Viability Assay

The non-fluorescent dye resazurin also identified as Alamar blue reagent (Sigma), is converted to red fluorescent resorufin via reduction reactions by metabolically active cells. The reagent was prepared in DMEM medium without FBS at a concentration of 1 µM and was directly added to the cells for 2 hours incubation. The fluorescence of each well was quantified in a plate reader (Envision Xcite, Perkin Elmer) with an excitation wavelength of 545 nm and an emission wavelength of 590 nm. Fluorescence was measured immediately after the addition of the reagent and after the 2 hours incubation. The resulting difference between these two fluorescence values was calculated.

### Library

A first screen was performed using the Human Endogenous Ligand Library (LIGENDO, Greenpharma, France) that presents a large molecular diversity of compounds defined as metabolite-like. Compounds were dissolved in dimethylsulfoxide (DMSO) at a concentration of 0.04 mg/ml. In first intention, we screened a selection of 68 compounds from this library. This library is composed of human ligands that are all believed to be biologically active. As this was a first trial aimed at validating the strategy, from our knowledge and data in the literature, we selected in this library compounds that could potentially be active on energy metabolism. These 68 compounds were chosen among the 420 compounds to avoid redundancy of molecular types and to ensure a large representation of potentially relevant metabolic pathways. To design a plate containing 80 compounds, we added 12 other compounds of potential interest (Table S2). The final dilution was 4.7 µg/ml in each 100 µl well.

### Total Respiration

After 7 days of differentiation, H9c2 cells were treated with the selected molecules in a differentiation medium supplemented with 0.1% BSA for 3 days. H9c2 cellular respiration was measured by using an oxygen-sensing Clarke electrode in a respiration medium after 5 minutes of digitonin permeabilization. This respiration medium contained (in mM): 2.77 CaK_2_ EGTA, 7.23 K_2_EGTA, 1.38 MgCl_2_, 3 K_2_HPO_4_, 20 imidazole, 20 taurine, 0.5 DTT, 90 K-methanesulfonate, 10 Na-methanesulfonate, 10 glutamate and 4 malate. Values are normalized to the protein content in each chamber. Proteins are measured using the bicinchoninic acid protein assay kit (Sigma).

### Adult Rat Ventricular Myocyte Isolation

Adult rat ventricular myocytes were isolated using retrograde perfusion of isolated heart with collagenase [23]. Freshly isolated cardiomyocytes were plated on laminin-coated culture dishes at a density of 2.10^5^ cells/dish in minimal essential medium (MEM from SIGMA) supplemented with 2.5% FBS with PS and 2% HEPES for 1 hour and then switched to serum-free medium for 15 hours. Cells were then treated with the selected compounds for 9 and 24 hours.

### LDH Activity Measurement

The compound toxicity was evaluated by measuring lactate dehydrogenase activity in cardiomyocyte culture medium using a biochemical assay with NADH and pyruvate. Measurements were performed in duplicate and read at a wavelength of 340 nm with a spectrophotometer (Uvikon xs, Bioserv).

### Statistics

All data are expressed as means ± S.E.M. A non parametric Mann and Withney test was used in all experiments to determine differences between conditions with significance set at p<0.05. For correlation, linear regression curve fit was used.

## Supporting Information

Table S1 **List of the 62 compounds from Ligendo tested in first intention.** This table represents the 80 compounds tested with molecule name, structure, molecular weight, assay concentration, metabolic pathways and fold induction in GLuc activity with the significance.
(DOC)

Table S2 **List of primers used for qPCR.** TBP indicates TATA binding protein; PGC-1α, peroxisome proliferator-activated receptor γ coactivator 1α; NRF-2, nuclear respiratory factor 2; Tfam, mitochondrial transcription factor A; COX I and COX IV, cytochrome c oxidase subunits I and IV; ERRα, estrogen related receptor α; PPARα, peroxisome proliferator-activated receptor α; MCAD, medium-chain acyl-coenzyme A dehydrogenase; PDK4, pyruvate dehydrogenase kinase 4; PGC-1β, peroxisome proliferator-activated receptor γ coactivator 1β.
(DOC)

Table S3 **List of the 25 compounds selected as “hits” positives both for GLuc activity and GLuc mRNA expression.** Results are expressed as fold induction both for GLuc activity (N = 4) and Gluc mRNA expression. *, p<0.05 and **, p<0.01.
(DOC)
