# Supplementary material for: A Cardiac-Specific Robotized Cellular Assay Identified Families of Human Ligands as Inducers of PGC-1α Expression and Mitochondrial Biogenesis
Source: PLoS One. 2012 Oct 3;7(10):e46753. doi: 10.1371/journal.pone.0046753 (PMC3463514; doi:10.1371/journal.pone.0046753)
Supplement: Table S1 — List of the 62 compounds from Ligendo tested in first intention. This table represents the 80 compounds tested with molecule name, structure, molecular weight, assay concentration, metabolic pathways and fold induction in GLuc activity with the significance. (DOC) [file pone.0046753.s001.doc]

**Supporting information**

**Table S1. List of the 62 compounds from Ligendo tested in first intention**

This table represents the 80 compounds tested with molecule name, structure, molecular weight, assay concentration, metabolic pathways and fold induction in GLuc activity with the significance.
