# Supplementary material for: A Cardiac-Specific Robotized Cellular Assay Identified Families of Human Ligands as Inducers of PGC-1α Expression and Mitochondrial Biogenesis
Source: PLoS One. 2012 Oct 3;7(10):e46753. doi: 10.1371/journal.pone.0046753 (PMC3463514; doi:10.1371/journal.pone.0046753)
Supplement: Table S2 — List of primers used for qPCR. TBP indicates TATA binding protein; PGC-1α, peroxisome proliferator-activated receptor γ coactivator 1α; NRF-2, nuclear respiratory factor 2; Tfam, mitochondrial transcription factor A; COX I and COX IV, cytochrome c oxidase subunits I and IV; ERRα, estrogen related receptor α; PPARα, peroxisome proliferator-activated receptor α; MCAD, medium-chain acyl-coenzyme A dehydrogenase; PDK4, pyruvate dehydrogenase kinase 4; PGC-1β, peroxisome proliferator-activated receptor γ coactivator 1β. (DOC) [file pone.0046753.s002.doc]

**Table S2. List of primers used for qPCR**

| Target name | Forward primer  Reverse primer (5' → 3') | Annealing temperature (° C) |
| --- | --- | --- |
| TBP | AAA GAC CAT TGC ACT TCG TG  GCT CCT GTG CAC ACC ATT TT | 60 |
| Gaussia Luciferase | CAC GCC CAA GAT GAA GAA GT  TTG TGC AGT CCA CAC ACA GA | 60 |
| PGC-1α | CAC CAA ACC CAC AGA GAA CAG  GCA GTT CCA GAG AGT TCC ACA | 58 |
| NRF2α | CAC CAC ACT CAA CAT TTC GG  CCT TGG GGA CCT TTG AAC TT | 58 |
| Tfam | GAA AGC ACA AAT CAA GAG GAG  CTG CTT TTC ATC ATG AGA CAG | 60 |
| COXI | AGC AGG AAT AGT AGG GAC AGC  TGA GAG AAG TAG TAG GAC GGC | 60 |
| COXIV | TGG GAG TGT TGT GAA GAG TGA  GCA GTG AAG CCG ATG AAG AAC | 58 |
| ERRα | TCA AGG AGG GTG TGC GTC TG  CTT GGC CCA GCT GAT GGT GA | 65 |
| PPARα | ATG AGT CCC CTG GCA ATG  GGC ATT CTT CCA AAA CGG | 58 |
| MCAD | CCG TTC CCT CTC ATC AAA AG  ACA CCC ATA CGC CAA CTC TT | 60 |
| PDK4 | CCT TCA CAC CTT CAC CAC AT  AAA GAG GCG GTC AGT AAT CC | 60 |
| PGC-1β | TGG AAA GCC CCT GTG AGA GT  TTG TAT GGA GGT GTG GTG GG | 60 |

TBP indicates TATA binding protein; PGC-1α, peroxisome proliferator-activated receptor γ coactivator 1α; NRF-2, nuclear respiratory factor 2; Tfam, mitochondrial transcription factor A; COX I and COX IV, cytochrome c oxidase subunits I and IV; ERRα, estrogen related receptor α ; PPARα, peroxisome proliferator-activated receptor α; MCAD, [medium-chain acyl-coenzyme A dehydrogenase](http://en.wikipedia.org/wiki/Medium-chain_acyl-coenzyme_A_dehydrogenase_deficiency) ; PDK4, pyruvate dehydrogenase kinase 4; PGC-1β, peroxisome proliferator-activated receptor γ coactivator 1β.
