# Supplementary material for: A Cardiac-Specific Robotized Cellular Assay Identified Families of Human Ligands as Inducers of PGC-1α Expression and Mitochondrial Biogenesis
Source: PLoS One. 2012 Oct 3;7(10):e46753. doi: 10.1371/journal.pone.0046753 (PMC3463514; doi:10.1371/journal.pone.0046753)
Supplement: Table S3 — List of the 25 compounds selected as “hits” positives both for GLuc activity and GLuc mRNA expression. Results are expressed as fold induction both for GLuc activity (N = 4) and Gluc mRNA expression. *, p<0.05 and **, p<0.01. (DOC) [file pone.0046753.s003.doc]

**Table S3. List of the 25 compounds selected as “hits” positives both for GLuc activity and GLuc mRNA expression**

Results are expressed as fold induction both for GLuc activity (N=4) and GLuc mRNA expression. *, p<0.05 and **, p<0.01.
